# Complex Biventricular Pacing - A Case Series

**DOI:** 10.1016/s0972-6292(16)30714-8

**Published:** 2014-01-01

**Authors:** Emily Catherine Hodkinson, Keith Morrice, William Loan, Jacob Nicholas, EngWooi Chew

**Affiliations:** Belfast City Hospital, Belfast Health and Social Care Trust, 51 Lisburn Road, Belfast, BT9 7AB

**Keywords:** Biventricular Pacing

## Abstract

It is established that cardiac resynchronisation therapy (CRT) reduces mortality and hospitalisation and improves functional class in patients with NYHA class 3-4 heart failure, an ejection fraction of ≤ 35% and a QRS duration of ≥ 120ms. Recent updates in the American guidelines have expanded the demographic of patients in whom CRT may be appropriate. Here we present two cases of complex CRT; one with a conventional indication but occluded central veins and the second with a novel indication for CRT post cardiac transplant.

## Introduction

We present two cases of successful biventricular pacing in patients with complex anatomy. The first is via an occluded superior vena cava, the second in a transplanted heart.

## Case One

A 67-year-old man presented with New York Heart Association (NYHA) class 3 heart failure symptoms, left bundle branch block and a first degree AV node block (PR= 400ms). Echocardiography demonstrated a dysynchronous and dilated left ventricle with an estimated ejection fraction of 20%.

**Figure 1:**
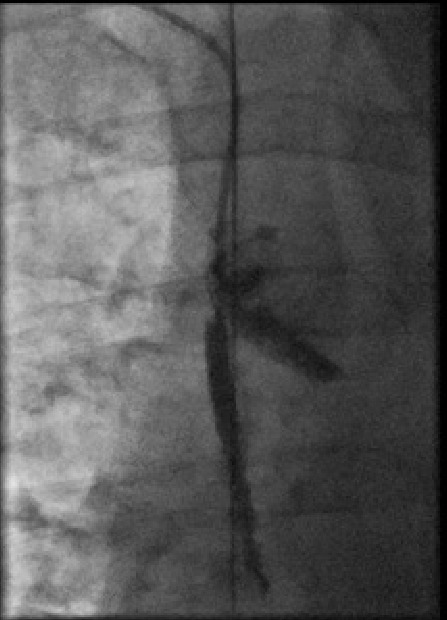
Superior Vena cava and Subclavian chronic total occlusions

His past medical history included dialysis-dependant renal failure and chronic lymphocytic leukaemia. Repeated cannulation of his central venous system had resulted in complete occlusion of the superior vena cava (SVC) and both subclavian veins (Figure 1). A functioning right internal jugular (IJ) tunnelled dialysis line was in-situ.

On the basis of comorbidities, he was deemed unsuitable for an epicardial left ventricular lead so he came forward for attempted transvenous approach.

**Figure 2:**
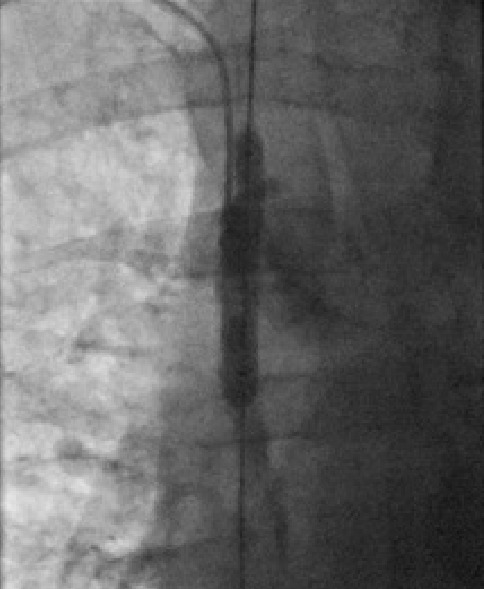
SVC balloon angioplasty

**Figure 3:**
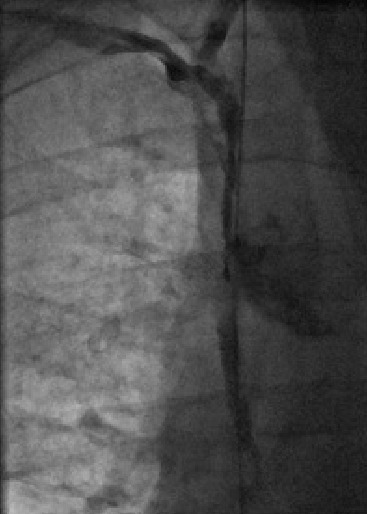
Successful recanalisation of the SVC and Subclavian veins

**Figure 4:**
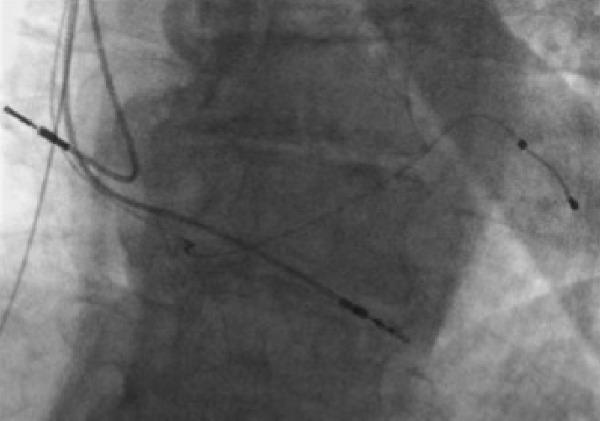
Final position of RV, LV and RA pacing leads

The IJ dialysis line was wired and the length of the track was sequentially dilated with 7mm and 9mm angioplasty balloons. The right subclavian vein was punctured under ultrasound guidance, wired with difficulty and eventually dilated up to 7mm (Figure 2). Recanalization of the SVC and right subclavian was achieved (Figure 3). An active fixation 6French bipolar pacing lead was sited in the right ventricular (RV) apex. A larger french defibrillation lead (for CRT-D) could not be accommodated. The coronary sinus was cannulated with a multipurpose shaped guiding catheter and a Medtronic Attain Ability® LV lead advanced to the terminal portion of a lateral vein. A 5Fr passive fixation pacing lead was advanced to the right atrium. A paediatric dialysis catheter was re-sited in the right IJ vein. The final lead positions were satisfactory (Figure 4).

The procedure was completed without complication. At 4 months, the patient's functional class was NYHA 2 with significant improvements in LV ejection fraction, mitral regurgitation and end diastolic dimensions (Table 1) demonstrated on echocardiography.

## Case Two

Patient Two, a 49-year-old male underwent orthotopic heart transplantation for end-stage ischaemic cardiomyopathy. He remained NYHA and Canadian Cardiovascular Society (CCS) class 1 for 10 years. Regular dobutamine stress echocardiography demonstrated good LV function and normal wall motion.

Twelve years post-transplant, he developed symptomatic high-grade atrioventricular block and a dual chamber St Jude Zephyr™ pacemaker was implanted. Unfortunately, significant and progressive left ventricular (LV) impairment developed soon after implant (Table 2). Rejection was excluded and a decision made to upgrade to a biventricular device.

An incision was made over the existing scar. Via a subclavian puncture, the coronary sinus was engaged with an AL3 diagnostic catheter and a Medtronic Attain Ability® LV lead was placed in a lateral branch of the coronary sinus with good stability and pacing indices. A Medtronic Syncra® CRT-P device was implanted. (the patient had declined a CRT-D device).

The patient improved almost immediately with an NYHA class of 1. Repeat echo at 3 and 6 months showed improvement in LV systolic function and reduction in wall hypertrophy (Table 2)

## Discussion

Central venous stenosis is common in dialysis patients. The exact prevalence is unclear but estimates range from 16 -50%. [1] Several techniques have been published in the literature to overcome the issue of venous occlusion in patients requiring pacing. Successful CRT via the ilio-femoral, [2] and internal jugular 3 route has been performed. Venoplasty has been shown to be useful in central venous chronic total occlusions allowing pacemaker upgrades as well as de novo implants. [4,5] Collateral veins can also act as a conduit to the right atrium in the case of occluded subclavian veins [6] and an 'inside-out' technique has been described where dilators are fed from the femoral vein, via the right atrium to the occluded subclavian. [7]

However, we are not aware of a case of de novo biventricular pacing via chronically occluded SVC and subclavian veins. It is also hoped that the recanalisation of both the SVC and the right subclavian veins will facilitate further insertion of dialysis cannulae if required.

The significant symptomatic and echocardiographic improvement in the patient with CRT is very encouraging, particularly given that CRT response rates in this group (dialysis dependant with associated malignancy) is very poor.

Late development of atrioventricular nodal block after cardiac transplantation is uncommon. It can be intermittent but is a definite indication for permanent pacing. [8] It is well established, however, that RV pacing-induced electrical and intra and interventricular mechanical dyssynchrony is a cause of heart failure [9,10]

There is evidence that upgrade of RV-only pacing systems to a biventricular device in patients with pacing-induced heart failure gain an improvement in LV function, NYHA functional class and a reduction in electrical and mechanical dyssynchrony [11-13]

Although the current UK guidelines for the use of CRT [14] (ejection fraction <35%, QRS >120msecs and NHYA symptoms 3 to 4) do not endorse the use of biventricular pacing in this patient population, we recommend that in heart transplant recipients who will require a higher percentage of RV pacing CRT be considered. Indeed the very recently updated ACC/HRS/AHA guidelines [15] on the appropriate use of ICD and CRT suggest that it may be appropriate to use biventricular pacing from the outset in patients with a pacemaker indication but preserved LV function in whom > 40% RV pacing is anticipated. We note a single case in the literature of CRT use post-transplant [16]

These cases demonstrate that with the correct equipment, team approach and careful clinical assessment, cardiac resynchronisation therapy can be successfully utilised in patients who would previously have been deemed unsuitable.

## Figures and Tables

**Table 1:**
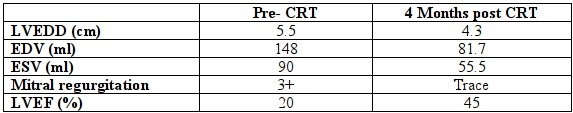
Echocardiography Pre- and post- Cardiac Resynchronisation therapy - Case 1

LVEDD= Left Ventricular End Diastolic Diameter, EDV=End Diastolic Volume, ESV= End Systolic Volume, LVEF = Left Ventricular ejection fraction, CRT = cardiac resynchronisation therapy

**Table 2:**
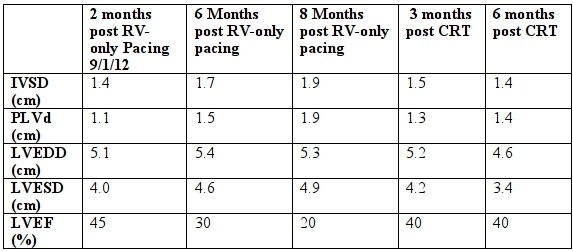
Echo parameters pre and post RV-only and Biventricular pacing

IVSD = Interventricular septal thickness in diastole, PLVD = Posterior left ventricular wall thickness in diastole, LVEDD= Left ventricular end diastolic diameter, LVESD= Left ventricular end systolic diameter, LVEF = Left Ventricular ejection fraction, RV = Right ventricular, CRT = Cardiac resynchronisation therapy

## References

[R1] Agarwal AK (2007). Central vein stenosis: a nephrologist's perspective. Seminars in dialysis.

[R2] Allred JD (2008). Biventricular ICD implantation using the iliofemoral approach: providing CRT to patients with occluded superior venous access. PACE.

[R3] Bosa-Ojeda F (2007). Upgrade of a pacemaker defibrillator to a biventricular device: the internal jugular vein approach in a case of bilateral subclavian veins occlusion. Journal of interventional cardiac electrophysiology.

[R4] McCotter CJ (2005). Placement of transvenous pacemaker and ICD leads across total chronic occlusions. PACE.

[R5] CHANGEME CHANGEME (2011). Percutaneous balloon venoplasty of pacemaker-associated superior vena cava obstruction to facilitate upgrade to a biventricular pacing system. Europace.

[R6] Brieda M (2011). Placement Of A Coronary Sinus Pacing Lead From A Sub-occluded Left Subclavian Vein Using A Collateral Vein To The Right Subclavian Vein. Indian pacing and electrophysiology journal.

[R7] Elayi CS (2011). Inside-out access: a new method of lead placement for patients with central venous occlusions. Heart rhythm.

[R8] Tay AE (2011). Permanent pacing for late-onset atrioventricular block in patients with heart transplantation: a single center experience. PACE.

[R9] Tops LF (2009). The effects of right ventricular apical pacing on ventricular function and dyssynchrony implications for therapy. J Am Coll Cardiol.

[R10] Brenyo A (2012). The downside of right ventricular apical pacing. Indian pacing and electrophysiology journal.

[R11] Delnoy PP (2009). Long-term clinical response of cardiac resynchronization after chronic right ventricular pacing. Am J Cardiol.

[R12] Dilaveris P (2006). Upgrade to biventricular pacing in patients with pacing-induced heart failure: can resynchronization do the trick? Europace : European pacing, arrhythmias, and cardiac electrophysiology : journal of the working groups on cardiac pacing, arrhythmias. and cardiac cellular electrophysiology of the European Society of Cardiology.

[R13] Horwich T (2004). Effects of resynchronization therapy on cardiac function in pacemaker patients "upgraded" to biventricular devices. J Cardiovasc Electrophysiol.

[R14] (2007). Cardiac resynchronisation therapy for the treatment of heart failure [Internet].

[R15] Russo AM (2013). ACCF/HRS/AHA/ASE/HFSA/SCAI/SCCT/SCMR 2013 Appropriate Use Criteria for Implantable Cardioverter-Defibrillators and Cardiac Resynchronization Therapy: A Report of the American College of Cardiology Foundation Appropriate Use Criteria Task Force, Heart Rhythm Society, American Heart Association, American Society of Echocardiography, Heart Failure Society of America, Society for Cardiovascular Angiography and Interventions, Society of Cardiovascular Computed Tomography, and Society for Cardiovascular Magnetic Resonance. J Am Coll Cardiol.

[R16] Apor A (2008). Successful cardiac resynchronization therapy after heart transplantation. Europace.

